# Opening Pandora's box?: ethical issues in prenatal whole genome and exome sequencing

**DOI:** 10.1002/pd.5114

**Published:** 2017-08-07

**Authors:** Ruth Horn, Michael Parker

**Affiliations:** ^1^ The Ethox Centre University of Oxford Oxford UK

## Abstract

**Objective:**

The development of genomic approaches to prenatal testing such as whole genome and exome sequencing offers the potential for a better understanding of prenatal structural anomalies in the fetus and ultimately for improved patient care and more informed reproductive decision making. In addition to the scientific and clinical challenges of achieving this, the introduction of new reproductive technologies also presents a number of ethical problems. The successful and appropriate development and introduction of prenatal genomics into clinical practice require these problems to be identified, understood and carefully analysed in the development of models of good ethical practice.

**Method:**

We conducted a critical review of the existing literature on ethical issues in prenatal genomics.

**Results:**

We identified and discussed five areas of particular concern: valid consent, management and feed‐back of information, responsibilities of health professionals, priority setting and resources and duties towards the future child.

**Conclusion:**

There is a need for further discussion of the issues we have outlined here, and we hope that this brief summary of ethical arguments in the literature encourages researchers, clinicians, patients and scientists to engage in further discussion of these and other important issues raised by prenatal genomics. © 2017 The Authors. *Prenatal Diagnosis* published by John Wiley & Sons, Ltd.

## Abstract 2

What's already known about this topic?

- There is a sizable literature on ethical issues in prenatal genetics. Yet, there is only limited literature discussing these issues in the particular context of prenatal genomics.

What does this study add?

- This literature review sheds light on the ethical issues in the particular context of prenatal genomics.
- It shows the need for further research in this area to provide an evidence base for the development, implementation and evaluation of models of good ethical practice.

## Introduction

Since the introduction of invasive testing for chromosomal anomalies in the mid‐1960s, costs have dropped and safety and accuracy have increased greatly.^1^ The development of new technologies such as whole genome microarrays with greater resolution enables the identification of still more anomalies with shorter turnaround times.^2^ Despite this progress, however, diagnosis is still only possible in around 40% of dysmorphic fetuses.^3^, ^4^ Prenatal whole genome and exome sequencing (WGES) offer the potential to provide diagnoses in cases where this is currently not possible.^5^ A good illustration of this potential is offered by the Prenatal Assessment of Genomes and Exomes project, which is analysing samples from 1000 parent–fetus trios in which structural abnormalities have been detected.^6^ The study is showing that prenatal genomic testing has the potential to increase diagnosis rates significantly over existing methods, and as techniques and approaches to analysis and interpretation improve, it is likely that this will improve still further, offering women and couples the opportunity to make better informed decisions about current or future pregnancies, and also ultimately informing the development of therapeutic interventions.^4^, ^7^

The development of non‐invasive prenatal testing (NIPT), which makes possible the analysis of cell‐free fetal DNA present in a sample of maternal blood (rather than through an invasive prenatal test), offers further important benefits.^8^ NIPT can be carried out earlier in pregnancy than existing invasive tests, and results are more accurate, which means invasive tests, with risk of miscarriage, are required less frequently.^9^ This also has the advantage that women who would not wish to undergo an invasive test but would accept a non‐invasive alternative would be able to receive information about their pregnancy that would not otherwise be available to them.^10^

Although the use of WGES in NIPT is in its early stages,^4^, ^11^ it is likely that it will soon be more widely available making it possible to know a great deal more about the genetics of a fetus from a maternal blood sample.^12^ This information will not only provide diagnosis but identify genetic variants indicating carrier status, late‐onset diseases and also, inevitably, information about variant that cannot currently be interpreted. As we will discuss below, the potential availability of each of these kinds of information raises important ethical questions.^13^

## Ethical Issues

Notwithstanding their important potential benefits, the use of genomic approaches to prenatal testing, particularly when combined with NIPT, raises a number of ethical questions that require careful consideration.^14^ Many of these have similarities with those discussed in the context of prenatal genetics.^15^, ^16^, ^17^, ^18^ However, in the context of prenatal genomics such issues arise in new ways, dimensions and in combinations not previously encountered.^13^, ^19^

### Achieving valid consent

Some of the most important ethical challenges in prenatal genomics relate to ensuring that patients have a good understanding of the implications of such testing before deciding whether or not to proceed. It is clearly important for genetic counsellors and professionals in fetal medicine to provide information in a way that is manageable and comprehensible.^20^ Clear communication about expectations and worries by adequately trained professionals are key.^21^ This is easier to say than to achieve, however, and its practical implementation will inevitably be difficult in many cases. There are several reasons for this. Some are to do with the complexity of the information involved and of the interpretation of results. Even where the result is agreed to be of clinical significance, its implications are likely to be difficult to explain and understand. This means that even in the most straightforward of cases, valid consent and the effective explanation of the relevance of findings may be difficult to achieve.

Still greater complexity arises from the fact that even where a variant is known to be significant, it is often not possible to know with certitude, in the prenatal context, whether the child will be affected by the disease. In many cases, moreover, notwithstanding the important progress being made in the field, much of the information resulting from genomic tests cannot yet be interpreted: the implications of genetic variants are often difficult to determine, and it is often unclear if a change in the genetic sequence is associated with an increased risk of a disease. Finally, the fact that sequencing can produce information (also of differing degrees of certainty with regard to the data interpretation) beyond what is relevant to the condition (the structural abnormality, say) under investigation adds a further layer of difficulty.

Against this background, a particularly important question is how much information about each of these possibilities needs to be provided at the time of consent and what levels of understanding are required for such consent (or refusal of consent) to be valid? The recent Montgomery ruling in the UK suggests that the threshold for information giving should be the information that this particular patient wants (complemented by consideration of what a prudent patient in this position might be expected to want).^22^ But how in the context of prenatal genomics ought this to be interpreted? In clinical genetics, a great deal of emphasis has tended to be placed on the notion of ‘consent as a process of communication’^23^, ^24^ in which ‘both clinicians and patients are seen as bringing information and values to the discussion’ and working together to agree on the information relevant to the patient.^25^ Clarke^23^ and Pinxten^24^ suggest that consent should be a ‘communicative process that is consent‐in‐action’ and an ‘opportunity to discuss the return of results in advance’ [^24^, p. 273], rather than a legalistic debate about consent forms [^23^, p.27]. Whilst of great value, however, this kind of conversation might be said to presuppose rather than provide an answer to the question of how much and what kind of information should be discussed, and as well as that of what constitutes best informational practice in consent to prenatal genomics.

Given that the concern with valid consent arises from recognition of the value of patient autonomy, it is noteworthy that it has sometimes been argued that in that giving parents, all available information may lead to ‘information overload and frustrate, rather than serve the aim of autonomous choice’[26, p.660]. This has sometimes been taken to justify ‘generic consent’ in which ‘an informed decision does not require individuals to be provided with all the information relevant to the decision in question […].’[27, p.1450–1451]. The argument here is that professionals should provide sufficient, yet filtered information to avoid information overload or ‘misinformed consent’.^28^ Elias and Annas^28^ argue that genetic counsellors and other professionals involved such as obstetricians should explain possible risks and problems using generic examples without however specifying all possible outcomes. One's first reaction to this model is likely to be that it is outdated and unduly paternalistic in the post‐Montgomery era. Nonetheless, it is clearly true that even a strongly autonomy‐driven, patient‐centred approach to information giving in prenatal genomics is going to need to involve the making of some prior decisions by health professionals about what information is to be offered and how. This suggests that a degree of paternalism is unavoidable.

### Managing and feeding back information

As indicated earlier, one of the most pressing practical ethical issues in prenatal genomics concerns the question of what information should be returned, to whom, by whom and when? Most discussions concern questions regarding the return of information about conditions that are preventable or treatable – ‘actionable’– conditions that are not actionable but clinically relevant or regarding the return of information about genetic variants with uncertain significance.

There is an emerging consensus in the ethics literature^24,29,30,31,31^ and professional guidelines^29^ that all data that are believed to be clinically ‘actionable’ should be fed back to patients or participants. This consensus is supported by empirical data showing that most people wish to receive information about those data whether it concerns the condition under investigation or an unexpected, incidental secondary finding.^30^, ^31^, ^32^ In the context of reproductive medicine, however, the concept of ‘actionability’ is likely to prove contested and value‐laden. Although some genomic variants are clearly defined as actionable, this is not always the case for all genomic variants with the potential to be actionable. In the prenatal context it is often difficult to determine whether a variant will affect the resulting child if the pregnancy were to be continued. Moreover, the question of whether a finding in prenatal testing is actionable is unlikely to be solely a scientific or clinical one. It is at least in part a matter of value and tied to questions of what is or is not a reasonable ground for termination of pregnancy and these are questions about which patients are likely to have their own views.

Moving beyond the question of what is or is not actionable, there is also much debate about the return of results which are clinically significant but ‘not directly actionable’ in the prenatal or paediatric context, such as those relating to adult‐onset diseases.^24^, ^33^, ^34^ A study by Kalynchuk et al.^35^ has shown that although information about adult‐onset diseases has the potential to trigger anxiety, the majority of parents still wish to be informed about such results whether the disease is treatable or not in order to make future pregnancy or other health decisions. Drawing upon his long experience in clinical genetics, Clarke calls for caution, however. He argues that parental knowledge about an otherwise healthy child's risk of an adult‐onset condition can sometimes lead parents to treat the child in ways that are harmful or overly restrictive: labelling the child as an ‘ill’ or ‘vulnerable’. In such cases, he argues, knowledge has the potential to be a burden both for the parents and for the child.^23^ Clearly, there is a need for careful thinking here. Another consideration that needs to be factored in is the fact that information from the fetus may be relevant to the parents themselves. If the testing reveals, for example, a BRCA mutation, the woman might benefit from screening and prophylactic surgery.

When it comes to information of uncertain significance, there are those who argue that data should not be returned even if patients would want this.^24^, ^33^ This view is particularly strong in research settings where the focus is on generalizable, population‐wide knowledge rather than on clinical treatment and decision making for an individual patient.^30^, ^36^ In some cases, the argument is made that the risk of misunderstanding the meaning of genetic variants with uncertain significance can generate unnecessary anxiety among tested persons.^37^, ^38^ Yet, it is important to note that several studies provide evidence that many participants would with to receive all genetic information relating to them, even that which is of uncertain significance. It appears that in some cases, such participants would prefer to be informed so that they can request and receive follow‐up from a genetic professional when more information about the variation is available, or participate in research.^39^ O′ Daniel and Hara point out that rather than generating unnecessary anxiety, offering participants the opportunity to receive all their genetic information, if they wish, may help building trust between the participants and those who offer such tests.^40^ Providing such information would inevitably present important demands on health resources, however, which itself raises important ethical questions. The opportunity costs of doing this would need to be carefully considered in any decision about which policy to adopt. It may be that this could be justified in cases where the variant of uncertain significance has a high chance of being pathogenic, and regular follow‐up will be important.

There is now agreement that information produced in clinical genomics should usually only be fed back where the patient has given consent for this. Much of this consensus emerged from the controversy following the publication in 2013 by the American College of Medical Genetics and Genomics of recommendations proposing that the feeding back of findings relating to mutations in 56 genes should be non‐optional.^41^ The American College of Medical Genetics and Genomics later revised their recommendations in line with guidelines elsewhere to acknowledge the importance of patient choice.^42^ Quite apart from the issue of respect for autonomy, Hall, Hallowell and Zimmern^43^ argue that the careful judging of benefits and harms needed calls for respect for parents' or patients' preferences and hence for their involvement.

One situation in which this likely to prove difficult is in relation to the possible or actual identification of misattributed paternity, where this is of clinical significance. What are the responsibilities of health professionals towards the woman, her partner(s) or the future child? Hercher and Jamal^44^ argue that ‘nondisclosure violates the norms of truthfulness and transparency that people have come to expect in medical settings. If discovered – and [in the genomic era] it is far more likely than ever before to be discovered – it will raise ethical and legal challenges.’ Lucassen and Parker^45^ also argue that the possibility of such information should be discussed in pre‐test counselling or when seeking consent. They argue that not informing the couple would be ‘unjustifiably paternalistic’. The implementation of any such policy would, however, require great sensitivity and would itself present a number of challenges.

### Responsibilities of health professionals

In addition to those discussed earlier, prenatal genomics presents important questions about the nature and scope of the responsibilities of health professionals to patients and families. Some of the most important of these arise out of the fact that current uses of prenatal genomics produce a great deal of information of uncertain significance whose meaning will likely become apparent over time as knowledge improves. In some cases, this will mean that diagnoses will be possible where this was not previously achievable. Is there in such situations a responsibility for health professionals and health systems to store and reanalyse data in the light of new knowledge? If such data are stored, is there a responsibility to recontact patients including any children? Clarke^23^ describes WGES data as a ‘lifetime resource’, but what are the obligations that this implies, and upon whom do such obligations fall?

A question of particular importance concerns the responsibilities to any child who results from a pregnancy in which genomic testing has been undertaken as he or she reaches maturity. Is there an obligation to inform the child about the test and its results? When should this happen? Is this the responsibility of parents, of health professionals or of some combination of the two? Thinking more broadly still, what are the obligations of health professionals (and families?) to act where information resulting from prenatal genomic test is of relevance and potential benefit to members of the patient's wider family? Should such information be treated as personal, confidential? Or should it be available for the care and treatment of other family members?^46^

### Priority setting and resources

All answers to the questions mentioned previously will have resource implications. More work is needed on the economic evaluation of genomics – ‘the real costs for the whole sequencing workflow, including data management and analysis, remain unknown’.^47^ It is clear, however, that the costs of data analysis, interpretation and curation are going to be significant – as are the costs of clinical time required to discuss findings with patients.^24^ This will remain the case even if the lower costs of sequencing mean that not all data need to be kept in expensive long‐term storage.^23^ In any publically funded health system, limits will have to be placed on the availability of prenatal genomics, and difficult decisions will need to be made about how to prioritise resources. Rogowski et al.^48^ argue that the criteria to be considered in order to justify the allocation of healthcare resources to genetic diagnostic testing should be the availability of effective treatment, the benefit of the diagnosis for the patient or the public welfare, the need of a diagnosis to facilitate decision‐making or the importance of the testing for research. But how are these different criteria to be judged against each other?

Those who are not offered genomic testing by the public sector but who nevertheless wish to receive their genomic information can, if they have the resources to do so, turn to commercial direct‐to‐consumer companies. This raises not only issues of equity (where those who get access are those who are able to pay) but also important issues about the regulation of standards of practice such as the quality of data interpretation and access to appropriate counselling.^49^, ^50^ The clinical value of WGES without medical indication remains unproven as the presence of a statistically significant variant does not mean that the person will be affected by this particular disease. Private companies that are sometimes based in different countries from where the tested person lives may not have the same standards of counselling explaining the meaning of the results.^51^ This is particularly challenging in the prenatal context where the phenotype of the child is not yet known. Furthermore, those accessing WGES privately may expect the public healthcare provider such as the NHS to support the interpretation of the test results and provide further intervention on the basis of these results. The interventions requested may not be in line with the healthcare providers' vision of good care and could put strain on an already overloaded healthcare system.^52^

### The future: is there a duty to have a healthy child?

Until recently, much of the ethical discussion about whether there is anything resembling a duty to have a healthy child, or at least a duty to avoid illness and disability where this is possible, and what this might mean, has focussed on the uses of preimplantation genetic diagnosis. However, it is likely as expertise and knowledge in both NIPT and genome editing and/or prenatal gene therapy increase and converge that questions about the responsibilities owed to future children in the context of a developing pregnancy are going to become matters for more mainstream discussion. For some, these issues will be largely unproblematic: where there is the potential to improve the health of a developing pregnancy at no risk to the mother or fetus, there will be an obligation to do so – a duty to ensure that any child has the best life possible.^53^, ^54^ For others, the possibility of such interventions will raise broader issues about the value of diversity, disability and questions about the importance of avoiding overly reductive conceptualisations of the ‘good life’. 55, 56, 57. Malek^53^ suggests that there is a difference between the wish to offer a child the best possible future and a discriminatory attitude towards existing persons with disabilities. Sparrow^58^ argues that the price of diversity carefully needs to be balanced with the well‐being of people. For others, these will be tied to questions about whether any developing child has a right to an open future and what this might mean?^59^ However, it might be argued by contrast that the right to an open future argument does not only concern questions about what parents should (not) do to their children but also what they ought to offer their children.^60^

## Conclusion

In this article, we have reviewed the literature relating to ethical issues arising in the use of genomic approaches to prenatal testing. Although there is a sizable literature on prenatal genetics, that on genomics is rather more limited, suggesting the need for further research in this area to provide an evidence base for the development, implementation and evaluation of models of good ethical practice. Perhaps the single biggest difference between genetics and genomics is likely to be the quantity and complexity of data generated and with it a proportionate increase in the challenges of analysis and interpretation and of translating this into meaningful information of use to health professionals and their patients. These challenges are amplified against the background of increasing sophistication of non‐invasive approaches and of the growth of commercial providers of such testing. It is our view that there is a pressing need for empirical research on and rigorous analysis of the practical ethical issues encountered by health professionals in the implementation of prenatal genomics, and on the experiences of patients and families. A further area in which more research would be welcome is on the role and uses of commercial companies in this space. There is a need for further discussion of the issues we have outlined here, and we hope that this brief summary of ethical arguments in the literature encourages researchers, clinicians, patients and scientists to engage in further discussion of these and other important issues raised by prenatal genomics.

**What's already known about this topic?**

- There is a sizable literature on ethical issues in prenatal genetics. Yet, there is only limited literature discussing these issues in the particular context of prenatal genomics.

**What does this study add?**

- This literature review sheds light on the ethical issues in the particular context of prenatal genomics.
- It shows the need for further research in this area to provide an evidence base for the development, implementation and evaluation of models of good ethical practice.
